# Biomechanical comparison of the undercut thread design *versus* conventional buttress thread for the lag screw of the dynamic hip screw system

**DOI:** 10.3389/fbioe.2022.1019172

**Published:** 2022-10-28

**Authors:** Fei Liu, Xiaoreng Feng, Jianxiong Zheng, Frankie Leung, Bin Chen

**Affiliations:** ^1^ Division of Orthopaedics and Traumatology, Department of Orthopaedics, Nanfang Hospital, Southern Medical University, Guangzhou, China; ^2^ Department of Orthopaedics and Traumatology, Yangjiang People’s Hospital, Yangjiang, China; ^3^ Department of Orthopaedics and Traumatology, Queen Mary Hospital, The University of Hong Kong, Hong Kong, Hong Kong SAR, China

**Keywords:** lag screw, dynamic hip screw, undercut thread, buttress thread, biomechanical test, finite element analysis

## Abstract

**Objective:** To compare the fixation stability of the lag screw with a undercut thread design for the dynamic hip screw (DHS) system *versus* the lag screw with the conventional buttress thread.

**Methods:** The lag screws with the undercut thread (a flat crest feature, a tip-facing undercut feature) and buttress thread were both manufactured. Fixation stability was investigated using cyclic compressive biomechanical testing on custom osteoporotic femoral head sawbone. The forces required for the same vertical displacement in the two types of lag screw were collected to evaluate the resistance to migration. Varus angle was measured on X-ray images to assess the ability in preventing varus collapse. Finite element analysis (FEA) was performed to analyze the stress and strain distribution at the bone-screw interface of the two types of lag screws.

**Results:** The biomechanical test demonstrated that the force required to achieve the same vertical displacement of the lag screw with the undercut thread was significantly larger than the lag screw with conventional buttress thread (*p* < 0.05). The average varus angles generated by the undercut and buttress threads were 3.38 ± 0.51° and 5.76 ± 0.38°, respectively (*p* < 0.05). The FEA revealed that the region of high-stress concentration in the bone surrounding the undercut thread was smaller than that surrounding the buttress thread.

**Conclusion:** The proposed DHS system lag screw with the undercut thread had higher migration resistance and superior fixation stability than the lag screw with the conventional buttress thread.

## 1 Introduction

Hip fractures are commonly encountered and challenging in the elderly population because of their poor bone quality (Veronese and Maggi, 2018; Dobre et al., 2021). Operation is usually necessary for this condition to decrease the complications associated with prolonged bed confinement (Roberts et al., 2015). The dynamic hip screw (DHS), which comprises a lag screw and a sliding plate, is a widely utilized fixation implant for hip fractures, especially for intertrochanteric hip fractures (Matre et al., 2013; Law et al., 2021; Lim et al., 2021). However, fixation failure of DHS is common. In particular, the failure incidence can reach 51.4% in unstable intertrochanteric hip fractures (Memon et al., 2021). Failure patterns include femoral head varus collapse, medialization of distal fragment and screw cut-out of the lag screw, which always result in a revision surgery (Boukebous et al., 2018; Taheriazam and Saeidinia, 2019; Memon et al., 2021).

Although fracture type and surgical techniques are crucial in fixation failure of DHS treatment (Hsu et al., 2016), the configurations of the lag screw are also critical factors; inner diameter, outer diameter, pitch, screw length and thread shape are main configuration parameters for orthopedics screws (Feng et al., 2015; Feng et al., 2016; Gustafson et al., 2019; Kilian et al., 2019; Schliemann et al., 2019). The thread shape is the most important structure, as it can determine the mechanical environment at the bone-screw interface, and influence the biomechanical performance and osseointegration, accordingly (Abuhussein et al., 2010; Alemayehu and Jeng, 2021; Feng et al., 2021; Watanabe et al., 2022). An FEA study found that under the same pullout force, the V-shaped thread and square thread generated less stress than the thin-thread at the bone-screw interface (Geng et al., 2004). The reverse buttress thread also exhibits superior performance in FEA and biomechanical pullout test (Gracco et al., 2012; Oswal et al., 2016). However, another biomechanical study showed that screws with trapezoidal thread had higher pullout strength than screws with reverse buttress thread (Yashwant et al., 2017). At present, the thread shape of the DHS lag screw is standard buttress thread, which was first proposed by Robert Danis in the 1950s and is prevalent among different orthopedics screws. The typical buttress thread has the optimal performance in terms of the resistance of the unidirectional axial pull-out loading force (Ricci et al., 2010). However, the loading forces are multiaxial during the physiological motion *in vivo*. The failure risk for standard buttress thread screw is high when exposed to multidirectional loading forces (Stahel et al., 2017). A clinical research and finite element analysis (FEA) validated that radial stress affects screw loosening in the plate fixation of long bone fractures more than axial stress (Feng et al., 2019). The major complication of DHS is screw cutout, usually due to lateral migration of the lag screw relative to the femoral head (Taheriazam and Saeidinia, 2019). Our previous researches have demonstrated that the undercut thread design for cancellous bone screw exhibited better lateral resistance than the standard buttress cancellous bone screw (Feng et al., 2022a). In addition, compared with the buttress thread, the undercut thread can make the stress distribution of the bone around the thread more uniform, and effectively avoid stress shielding, thus facilitating the process of osseointegration (Feng et al., 2022b).

The purpose of the present study was to evaluate the biomechanical performance of the DHS lag screw with the undercut thread design *versus* the standard conventional thread pattern (buttress thread) using biomechanical testing on surrogate femoral heads and FEA.

## 2 Materials and methods

### 2.1 Biomechanical test

**FIGURE 1:**
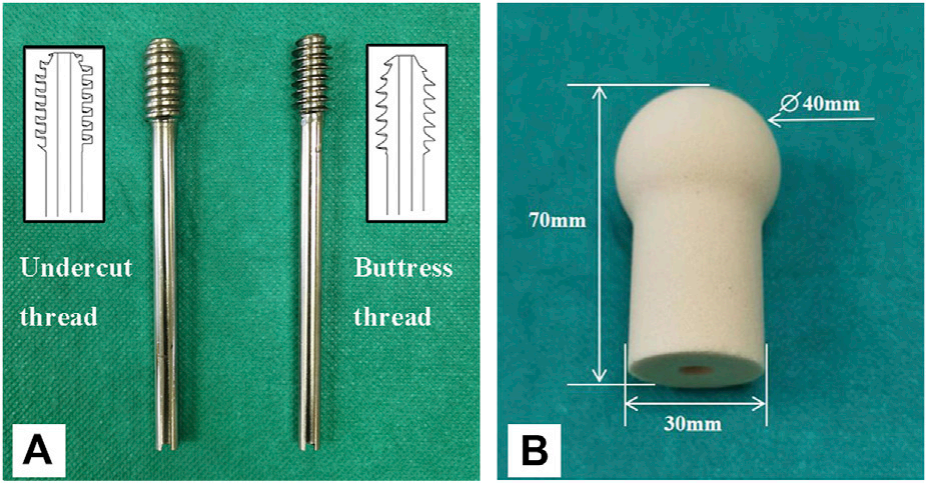
The lag screws and surrogate femoral head used in this study. **(A)** A lag screw with the undercut thread (left), and a lag screw with conventional buttress thread (right). **(B)** Surrogate femoral head manufactured from polyurethane foam blocks.

The DHS lag screws with undercut (*n* = 5) and buttress threads (*n* = 5) were manufactured from 316 low carbon vacuum melt stainless steel. Configurations of the screws were all identical except thread shape, including major diameter of 12.88 mm, minor diameter of 7.80 mm, thread pitch of 3.00 mm and screw length of 120 mm. Additionally, the undercut thread had a flat crest of 1.57 mm (Figure 1A).

**FIGURE 2:**
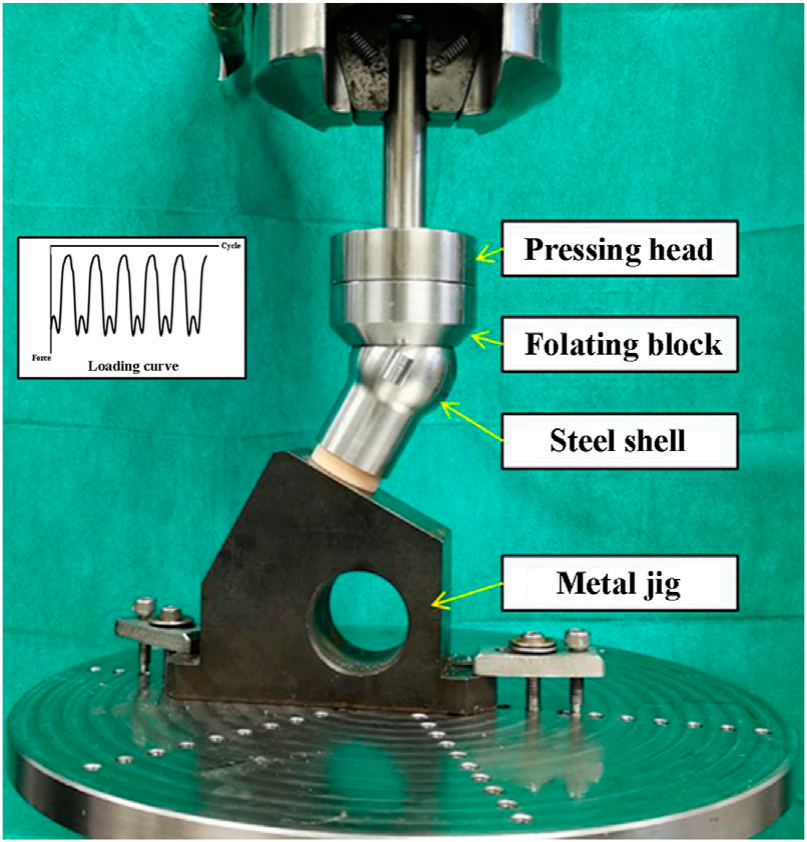
Setup of the biomechanical test of surrogate femoral head fixed with the lag screw.

Solid rigid polyurethane foam blocks with a density of 0.16 g/cm^3^ (Sawbones 10 PCF; Pacific Research Laboratories, Vashon, Washington, United States ) were used to imitate the human osteoporotic cancellous bone (O'Neill et al., 2012). The foam blocks were custom manufactured into the shape of a femoral head (Figure 1B). A pilot hole with the diameter of 8 mm and depth of 55 mm was reamed for each surrogate femoral head. Dimensions of the femoral head are shown in Figure 1B (Lee et al., 2014). A steel shell with a thickness of 2 mm that matched the shape of the femoral head was also custom manufactured (Figure 2). A metal jig was custom manufactured to fix the implant, with a special design slope to simulate the 17° clinical angle of peak walking force vector acting toward the center of the femoral head in the frontal plane (Figure 2) (Bergmann et al., 2016).

**FIGURE 3:**
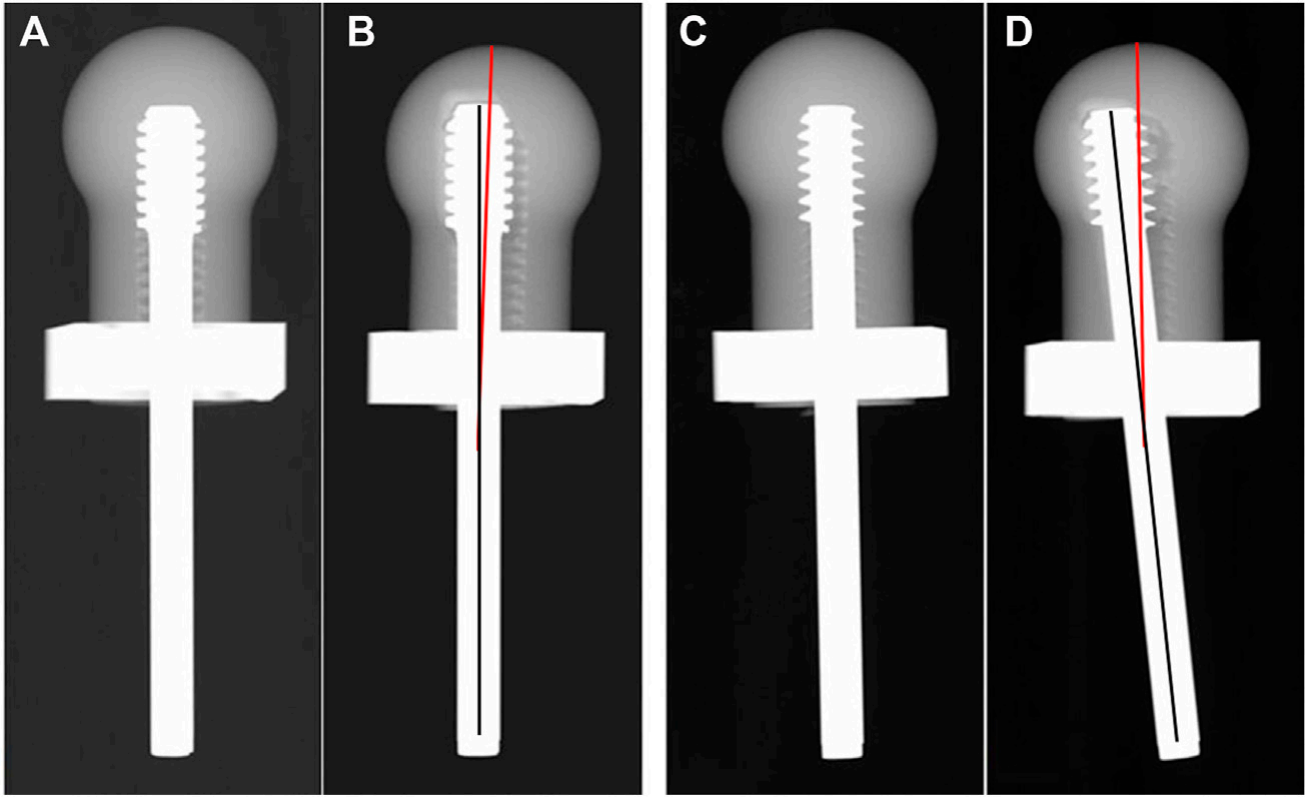
Images of X-ray scan pre-test **(A,C)** and post-test **(B,D)**. The solid black line represents the axis of the screw, the solid red line represents the axis of the femoral head.

Each lag screw was inserted into the pilot hole of the surrogate femoral head using a screwdriver. Digital X-ray images (20 kV, 5 s) were taken to ensure that the screw reached the end of the hole (Figure 3). Then, the femoral head was assembled with the steel shell and fixed into the metal jig. Subsequently, each sample was mounted on the loading cell of an MTS 858 Mini Bionix (MTS Systems Corporation, Eden Prairie, Minnesota, United States ) hydraulic loading machine for cyclic loading test (Figure 2). The loading force was applied to the steel shell through a pressing head and a floating block (Figure 2). The cyclic loading program was a double-peak loading curve with the minimum and maximum forces ranging from 100 N to 1000 N, respectively, for the first cycle. The maximum force increased by 0.2 N per cycle at 1 Hz with the minimum force kept at 10% of the maximum force. Continuous loading was applied until the vertical displacement of 7 mm was reached. After loading, a digital X-ray image was taken for each sample in the anterior-posterior (AP) view.

The load required to reach the axial displacement of 1, 2, 3, 4, 5, 6 and 7 mm was collected, respectively. The varus angle, which was defined as the angle between the axis of the lag screw and the central line of the femoral head after loading, was measured on the X-ray image in the AP view (Figure 3).

### 2.2 Finite element analysis

**FIGURE 4:**
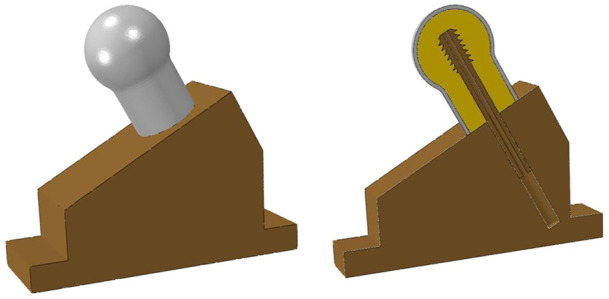
FEA model assembly. The right graph shows the mid-sagittal plane of the FEA models.

FEA is an engineering tool for structural analysis and has been widely used to evaluate the stress distribution of bones and implants. FEA can reveal detailed information, such as stress and strain distributions, that can be difficult or impossible to measure in laboratory biomechanical tests. The FEA models in this study were established to simulate the scenario of a human in a stationary standing position. The FEA 3D models included the two kinds of lag screws with different thread shape, the femoral head of the cancellous bone and the metal jig, which possessed the same dimensions as described previously in the biomechanical test section. The 3D model of cortical bone was designed as a shell with a thickness of 2 mm wrapped around the surface of the femoral head cancellous bone. The assembly of the 3D models is shown in Figure 4.

The 3D finite element models were created for each geometry using ABAQUS software suite (6.13/CAE; Simulia, Providence, Rhode Island, United States ). The metal jig was defined as a rigid body. The material properties of the cancellous bone, cortical bone, and screw are summarized in Table 1 (Benli et al., 2008; Saba, 2012; Lee et al., 2016). The material properties were all defined as homogeneous, isotropic and linear elastic (Feng et al., 2022a). The cancellous bone and screw were simulated as osteoporotic cancellous bone and stainless steel, respectively (Benli et al., 2008; Oftadeh et al., 2015; Lee et al., 2016). Bone-screw contact interfaces were defined as sliding interactions with a Coulomb friction coefficient of 0.3 (MacLeod et al., 2012). Bone-metal jig and screw-mental jig contact interfaces were defined as frictionless sliding interactions (Afferrante and Ciavarella, 2006). Cortical bone was tied with cancellous bone (Fu et al., 2022). A constant load of 250N was applied straight down on the femoral head with the bottom of the metal jig fixed (Ching et al., 2020).

**TABLE 1:** Material properties for the FEA models used in this study.

Material	Young’s modulus, MPa	Poisson’s ratio
Cancellous bone	260	0.29
Cortical bone	2600	0.3
Screw	200000	0.3

Since the stress distribution and strain distribution in the bone were the focus of this study, the bone was modeled using quadratic tetrahedral elements, while the screws and metal jig were modeled using linear tetrahedral elements. The approximate number of elements used in the cancellous bone, cortical bone, screw, and metal jig were 760,000, 120,000, 190,000, and 1,280,000, respectively. All bone models incorporated mesh refinement at the bone-screw interface. The size of the element edge near the screw hole was 0.02 mm. A mesh convergence study was performed to determine the appropriate mesh size for each part of the models based on their effect on the maximum Von Mises stress. The maximum Von Mises stress of the undercut thread model changed by 0.97%, when the number of elements in the bone was doubled. Therefore, the number of elements mentioned previously was considered suitable for the analysis of the FEA models used in this study.

To assess the effect of the undercut thread and the buttress thread on the bone, the maximum Von Mises stress, the maximum principal strain, and the minimum principal strain were evaluated for cancellous bone surrounding the bone-screw interface for the two FEA models in the mid-sagittal plane. In addition, the volume of the bone around the screw thread at the bone-screw interface, when the Von Mises stress exceeded a set threshold, was calculated for both FEA models.

## 3 Data and statistical analysis

The varus angle, and the load required to reach the axial displacement of 1, 2, 3, 4, 5, 6 and 7 mm were expressed as means and standard deviations (SD). An independent sample *t* test was used to compare differences between the two studied groups and *p* < 0.05 was considered statistically significant. All statistical analyses were conducted using SPSS (IBM SPSS Statistics for Windows, IBM Corp, Armonk, NY, Version 19.0).

## 4 Results

### 4.1 Biomechanical test

**FIGURE 5:**
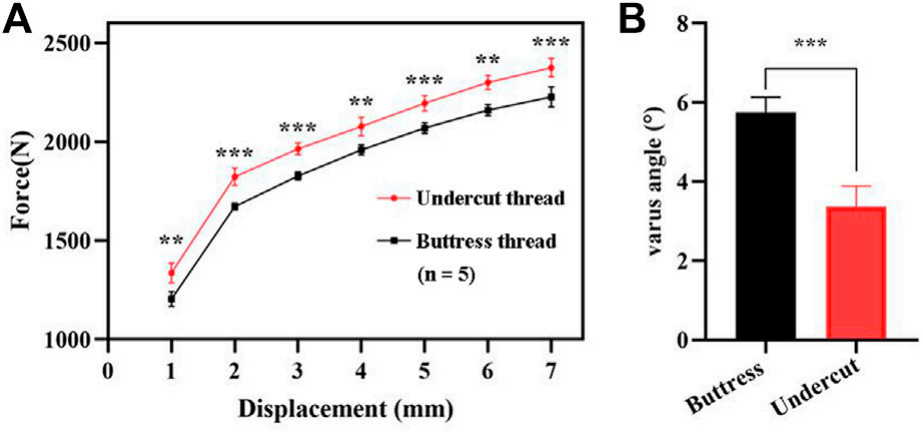
Results of the biomechanical test. **(A)** Force required to reach the vertical displacement of 1, 2, 3, 4, 5, 6, and 7 mm. **(B)** Varus angle generated post-test by the two types of lag screws. **, *p* < 0.01; ***, *p* < 0.001.

The mean load required to reach the axial displacement of 1, 2, 3, 4, 5, 6, and 7 mm for the proposed lag screw with undercut thread was 1,336.50 ± 49.58 N, 1882.67 ± 44.53 N, 1964.87 ± 29.63 N, 2078.55 ± 47.69 N, 2194.86 ± 39.32 N, 2300.47 ± 36.47 N, and 2375.29 ± 46.29 N, respectively. The corresponding mean load for the lag screw with buttress head was 1205.17 ± 38.25 N, 1672.07 ± 18.08 N, 1827.16 ± 20.05 N, 1959.64 ± 25.70 N, 2070.35 ± 27.25 N, 2161.02 ± 28.37 N, and 2227.88 ± 50.91 N, respectively (Figure 5A). The mean varus angles of the undercut and buttress thread lag screws were 3.38 ± 0.51° and 5.76 ± 0.38°, respectively (Figure 5B).

### 4.2 Finite element analysis

**FIGURE 6:**
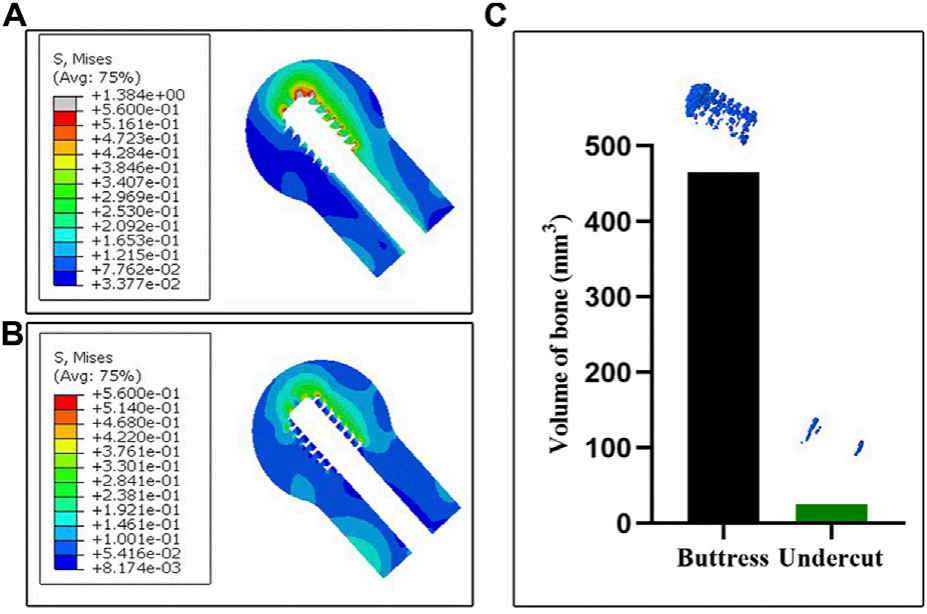
FEA results of the two models. **(A,B)** The Von Mises stress distribution of the cancellous bone in the mid-sagittal plane associated with the buttress thread and undercut thread, respectively. **(C)** The volume of bone with Von Mises stress >0.45 MPa.

The Von Mises stress distribution of the cancellous bone for the two FEA models in the mid-sagittal plane is illustrated in Figures 6A,B. The maximum Von Mises stresses of the undercut thread model and buttress thread models were 0.56 MPa and 1.38 MPa, respectively. Stress concentrations were observed at the superior screw-bone interface for both models. However, the area and value of the stress concentration of the undercut thread model were smaller than in buttress thread model. A threshold was defined as the 80% of the maximum Von Mises stress of the undercut thread model, which was 0.45 MPa. The volumes of the bone with Von Mises stress >0.45 MPa of the undercut thread model and buttress thread model were 25.63 mm^3^ and 465.30 mm^3^, respectively (Figure 6C).

**FIGURE 7:**
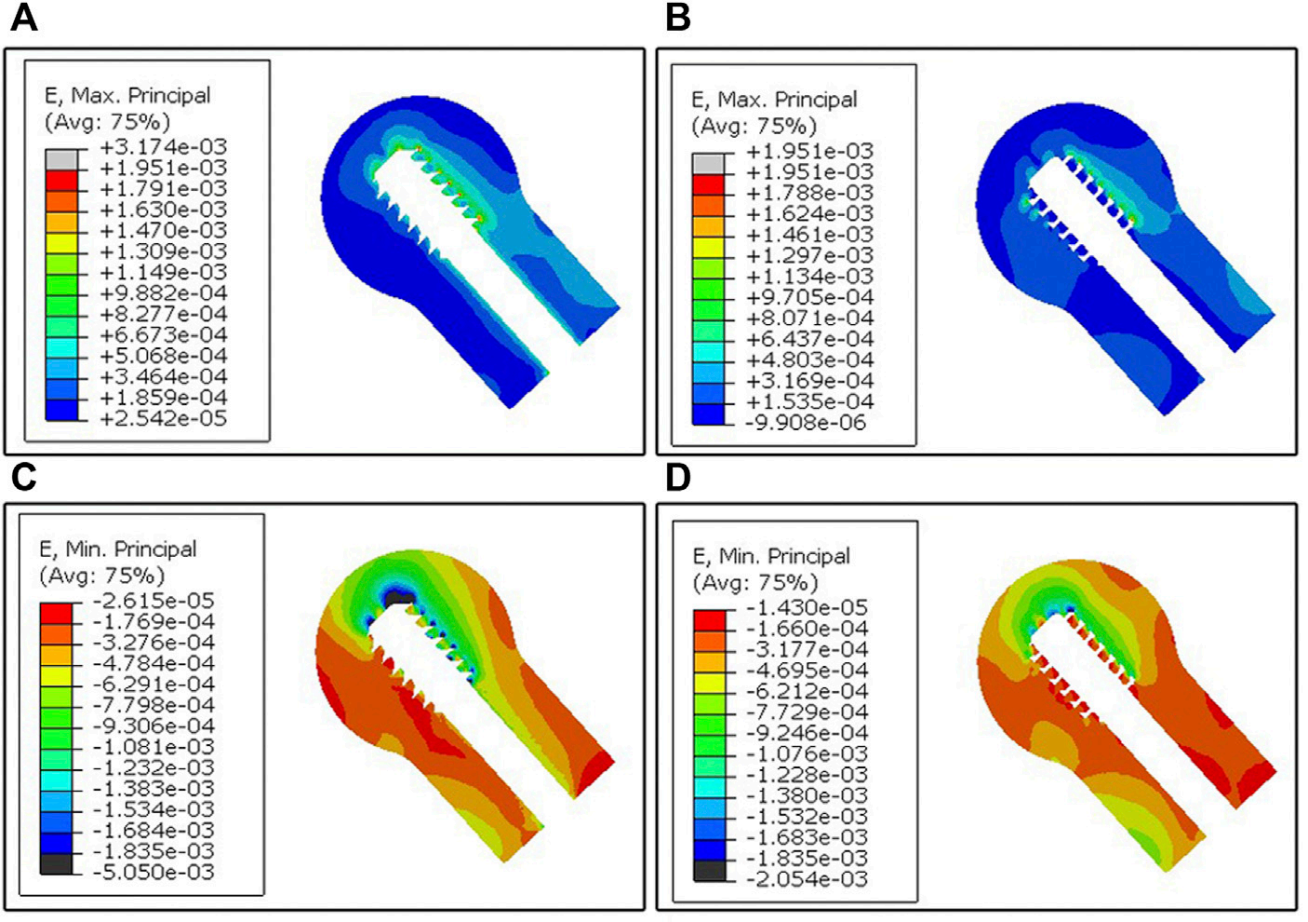
FEA results of the two models. **(A,B)** The maximum principal strain distribution of the cancellous bone in the mid-sagittal plane generated from the buttress thread and undercut thread, respectively. **(C,D)** The minimum principal strain distribution of the cancellous bone in the mid-sagittal plane generated from the buttress thread and undercut thread, respectively.

The maximum principal strain distribution of the cancellous bone in the mid-sagittal plane of the two femoral head models reveals the tensile strain distribution (Figure 7). The region of the tensile strain distribution concentration was observed at the superior screw-bone interface for both models. The area and value of the tensile strain distribution concentration of the undercut thread model were smaller than in the buttress thread model.

The minimum principal strain distribution of the cancellous bone in the mid-sagittal plane of the two femoral head models reveals the compressive strain distribution (Figure 7). The region of the compressive strain distribution concentration was also observed at the superior screw-bone interface for both models. The area of compressive strain distribution of the buttress thread model was larger compared to the undercut thread model.

## 5 Discussion

The results of the biomechanical test showed the proposed the undercut thread produced a higher load and a smaller varus angle when loaded to the same vertical displacement as the conventional buttress thread (Figure 5), which demonstrated the superiority of the resistance to migration of the proposed DHS lag screw with the undercut thread compared to the conventional buttress thread under the simulated physiological loading pattern. FEA revealed that the undercut thread can reduce the stress and strain of the cancellous bone at the bone-screw interface (Figures 6, 7). These results show that the undercut thread can potentially reduce the failure incidence of the DHS when treating hip fractures.

Although the biggest advantage of the buttress thread is the resistance to axial pullout forces (Ryu et al., 2014; Mosavar et al., 2015), bones *in vivo* need to withstand complex forces in multiple directions during physiological activities. A clinical investigation combined with FEA simulation demonstrated that axial stress was not the main reason for screw loosening (Feng et al., 2019). In this study, screw loosening was observed using X-ray images, which showed that the screw closest to the fracture line was most prone to loosening. FEA simulation indicated the radial stress, rather than the axial stress, produced more volume of bone destruction, which induced bone resorption and in turn led to screw loosening. The buttress thread design screw can produce microfractures at the bone-screw interface, as it is more likely to stripping (Stahel et al., 2017). Previous studies have validated that the undercut thread can effectively reduce stress concentration and stress shielding and that it possesses superior fixation stability in both lateral and torsional loading (Feng et al., 2022a; Feng et al., 2022b). In the present study, the undercut thread exhibited higher resistance to migration, compared to the buttress thread, in the biomechanical test simulated physiological load of the hip joint. FEA results suggested that the reason for this phenomenon could be the undercut thread that can reduce the Von Mises stress at the bone-screw surface. The peak Von Mises stress was 1.38 MPa for the buttress thread, whereas for the undercut thread, it was 0.56 MPa. The volume of the bone with stress higher than 0.45 MPa of the buttress thread model was larger than that of the undercut thread model, which meant that less bone destruction would occur surrounding the undercut thread. The flat crest of the undercut thread increased the lateral surface area of the thread meant that larger force was needed to achieve the same displacement as the buttress thread. This is the theoretical reason for the lower bone-screw interface stress of the undercut thread. In other words, the fixation construct with the undercut thread lag screw had greater stiffness and was stabler than the buttress thread under cyclic loading.

Varus collapse of the femoral head is the most common failure of DHS, often accompanied with cut-out of the lag screw through the femoral head, which usually requires revision surgery treatment, like total hip arthroplasty (Chen et al., 2017; Taheriazam and Saeidinia, 2019). In this study, the degree of varus collapse can be represented by the varus angle. In the case of the undercut thread, the mean varus angle after the biomechanical test was 3.38° ± 0.51°, which was significantly smaller than for the buttress thread with 5.76° ± 0.38°. The results demonstrated the undercut thread could effectively reduce the incidence of the varus collapse and cut-out of the lag screw for DHS treatment. Larger varus angles can result in greater deformation of the surrounding cancellous bone at the bone-screw interface. Consequently, increased volume of bone destruction and resorption is produced. This was revealed by the features of the post-test X-ray images and FEA simulation. Compared with the undercut thread, X-ray images showed that more cancellous bone was stripped by the buttress thread. The FEA simulation revealed that the maximum and minimum principal strain was larger at the bone-screw interface of the buttress thread than in the undercut thread. In other words, the undercut thread can reduce the microfracture occurrence in the cancellous bone at the bone-screw interface and thus lower the incidence of varus collapse.

Loads applied to the bone-screw interface can produce three types of forces on the surrounding bone tissue, including compressive, tensile and shear forces (Misch and Abbas, 2008). Bone tissue is more resistant to compression than tension (Oftadeh et al., 2015; Morgan et al., 2018). Compressive force is the most beneficial to bone tissue as it can increase bone density and its strength (Bolind et al., 2005). On the other hand, shear force could impair the mechanical properties of the trabecular bone (Wang and Niebur, 2006; Misch and Abbas, 2008). Different thread shapes have a significant effect on the distribution and proportion of these three types of forces (Abuhussein et al., 2010). Conceptually, the flat crest of undercut thread can reduce the shear stress and increase the proportion of compressive stress compared to the buttress thread, when subjected to lateral loads. Therefore, the mechanical environment at the bone-screw interface by the undercut thread design is favorable to bone resorption reduction and osseointegration improvement. However, this structure increases the lateral area of the thread, which may cause difficulty in screwing in. Therefore, in the clinical scenario, the screw with this thread may need a self-tapping structure.

Limitations exist in this study. Polyurethane foam of the Sawbones synthetic bone is a homogeneous material and cannot accurately reflect the reality in which cancellous bone is highly heterogeneous *in vivo*. Thus, conclusions based on results of the biomechanical test on artificial bone should be cautious. The models used were not an intact femur, so the conclusions may not represent the real clinical situation. Therefore, biomechanical testing on the cadaveric specimen should be conducted to validate the conclusions of this study in the future.

## 6 Conclusion

Overall, the lag screw of dynamic hip screw with the undercut thread had higher resistance to migration than the lag screw with conventional buttress thread. Biomechanical testing demonstrated that the lag screw with the undercut thread required a higher force to reach the same displacement as the lag screw with the buttress thread. Furthermore, the varus angle generated by the undercut thread was much smaller. Finite element analysis revealed that the Von Mises stress, the maximum and minimum principal strain at the bone-screw interface around the undercut thread were lower. The undercut thread can effectively relieve the high-stress concentration compared with the buttress thread.

## Data Availability

The raw data supporting the conclusion of this article will be made available by the authors, without undue reservation.
